# Supplementary figures and images for: Mapping mitonuclear epistasis using a novel recombinant yeast population
Source: PLoS Genet. 2023 Mar 29;19(3):e1010401. doi: 10.1371/journal.pgen.1010401 (PMC10085025; doi:10.1371/journal.pgen.1010401)

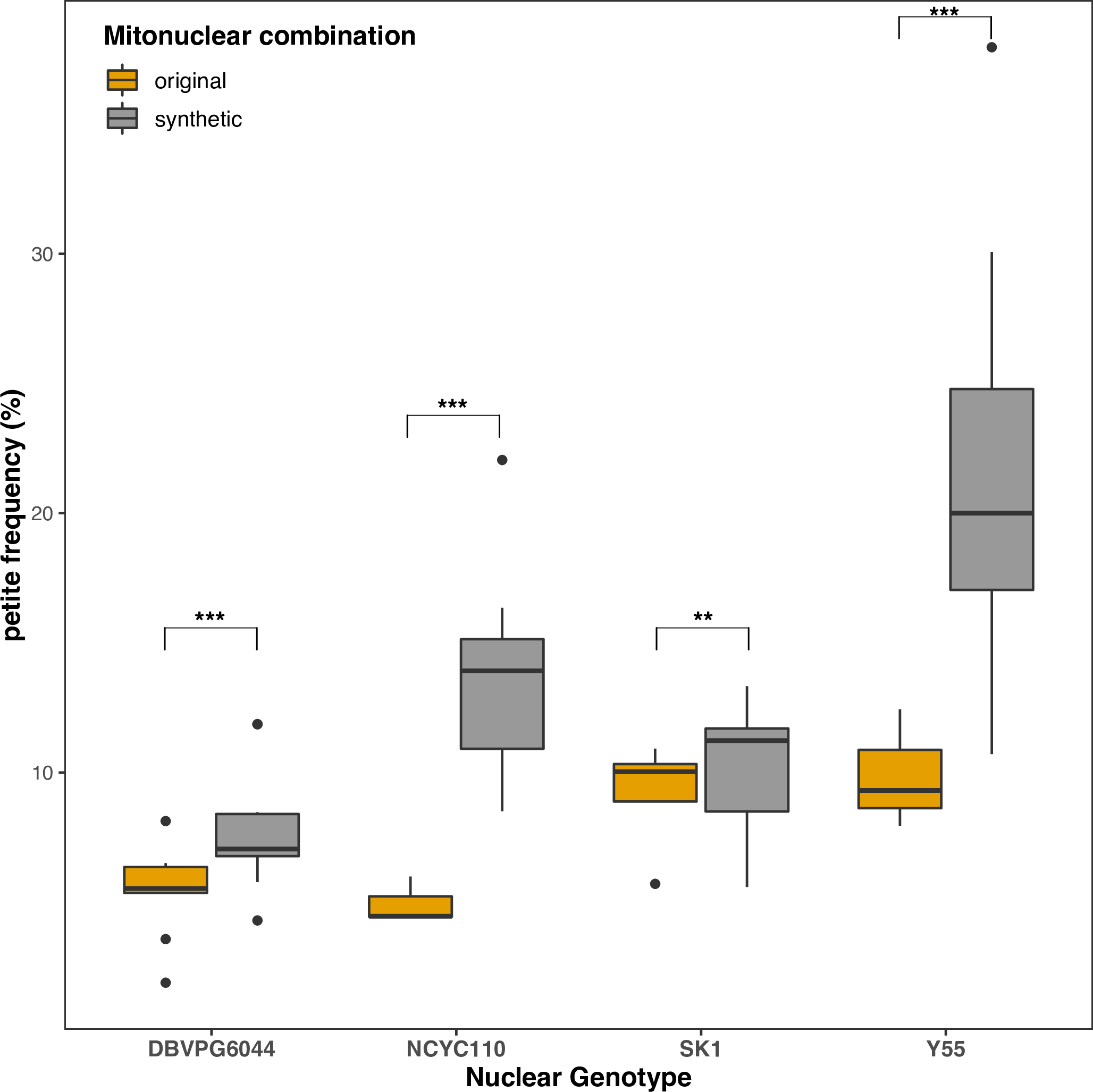

Supplement: S1 Fig — Petite frequencies of strains with the original (gold) vs. synthetic (grey) mitonuclear genotypes from Fig 3 are replotted as box plots with the synthetic combinations combined. All nuclear and mtDNAs are from strains with West African lineages. ANOVA significances are shown. * P<0.05, ** P ≤ 0.005, *** P ≤ 0.001. (TIF) [file pgen.1010401.s016.tif]

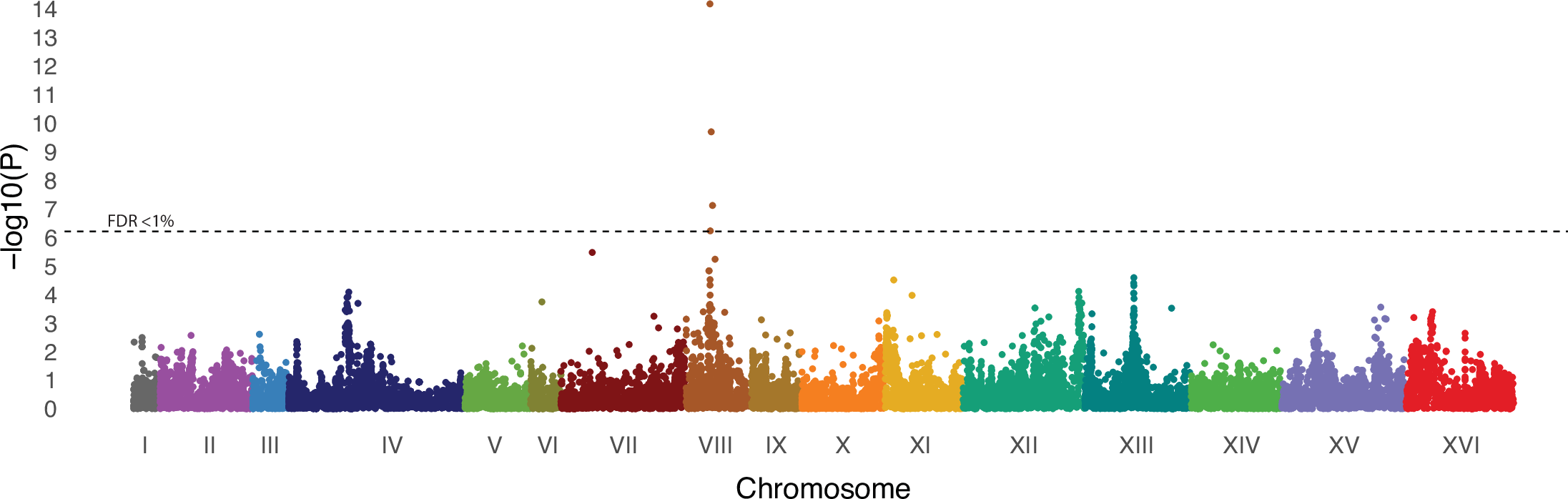

Supplement: S2 Fig — A Manhattan plot of -log10 of P values plotted against chromosomal position shows associations for maximal colony sizes for RC1 strains grown on copper sulfate. A single peak, corresponding to the location of CUP1 on Chr. 8, is the only significant association at FDR<1%. (TIF) [file pgen.1010401.s017.tif]

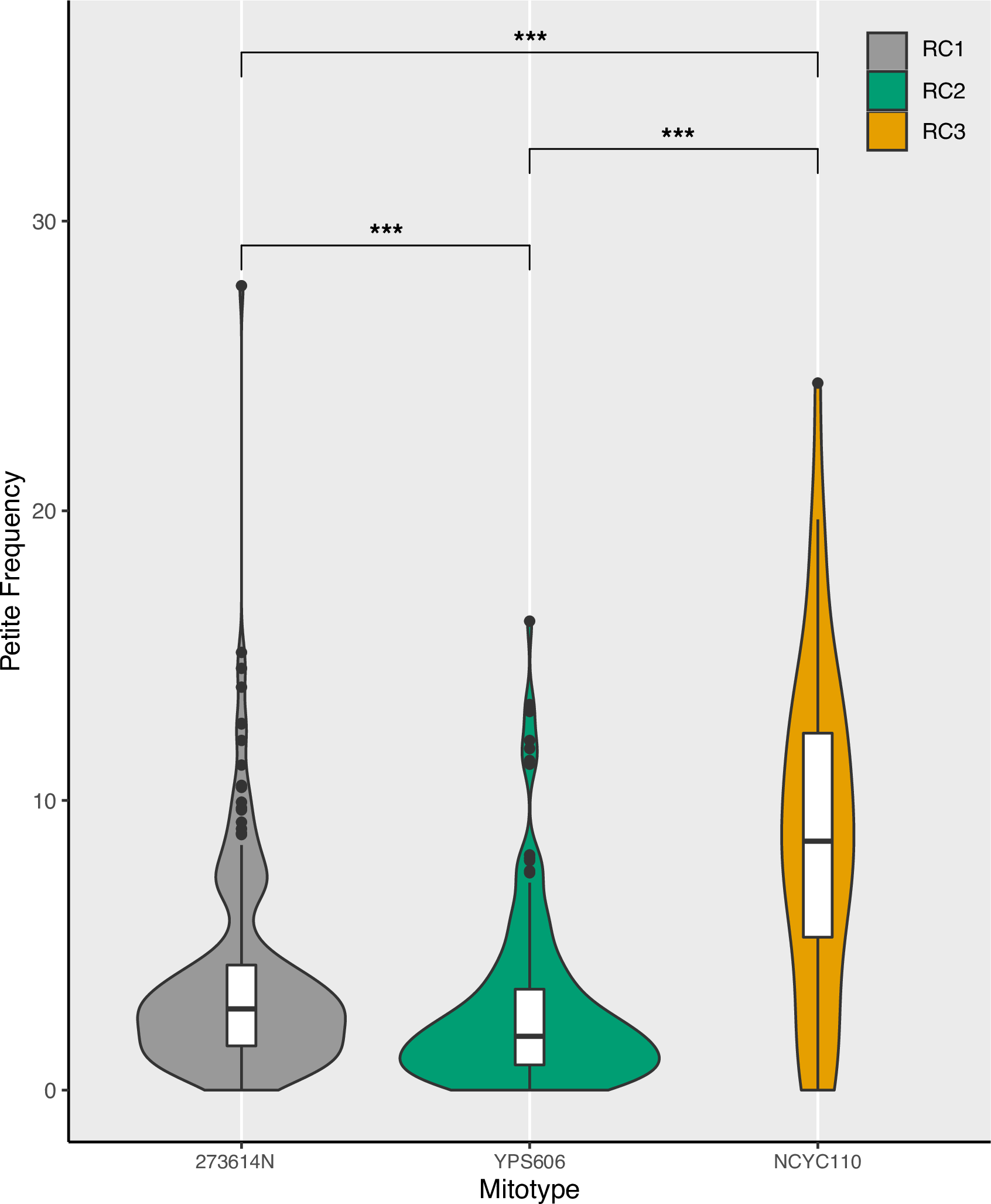

Supplement: S3 Fig — Petite frequencies of strains from RC1, RC2, and RC3 are presented as violin plots. RC1 strains harbor mtDNA from 273614N containing a medium number (137) of GC-clusters. RC2 strains harbor mtDNA from YPS606 containing a low number (117) of GC-clusters. RC3 harbor mtDNA from NCYC110 containing a high number (210) of GC-clusters. (TIF) [file pgen.1010401.s018.tif]

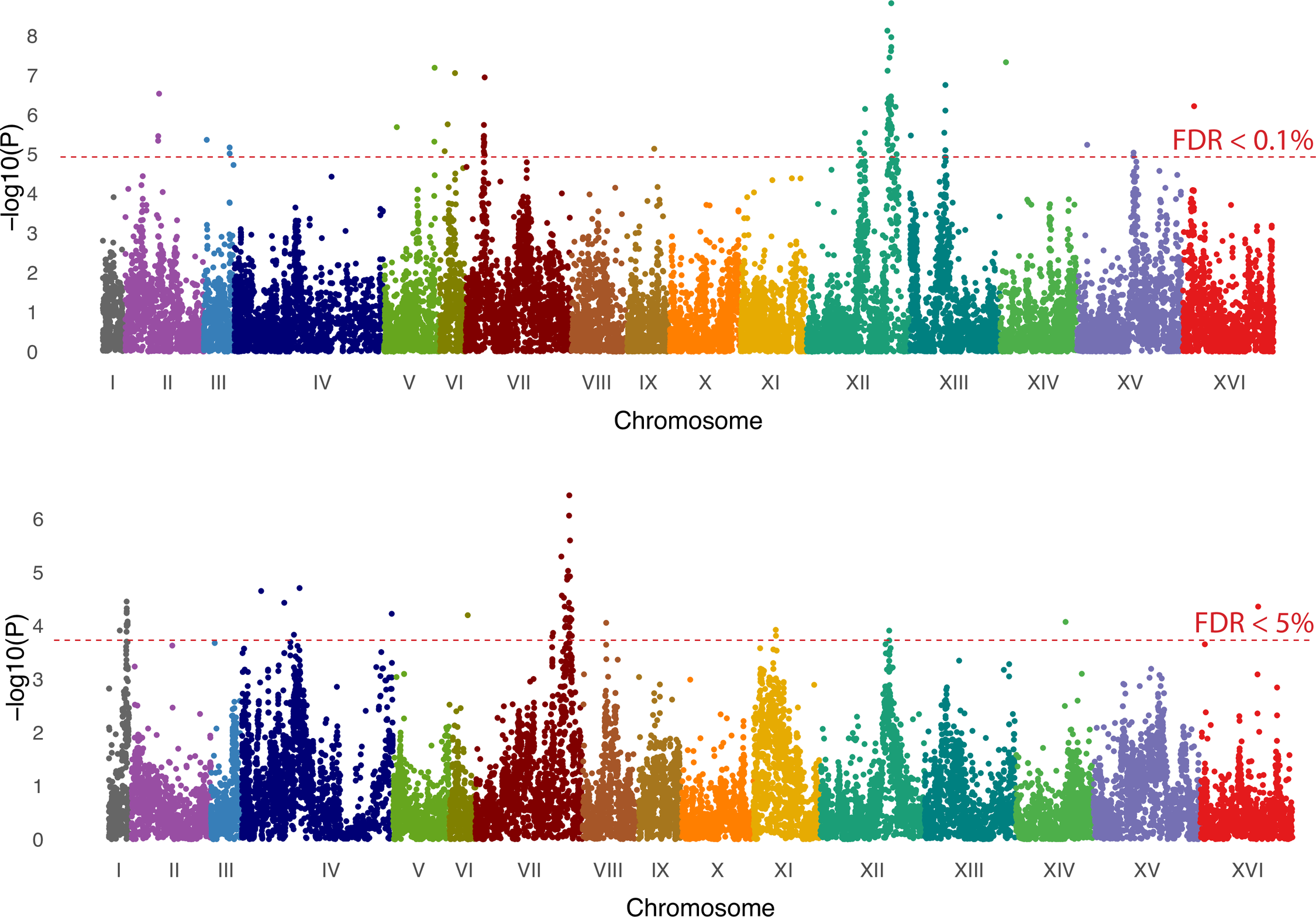

Supplement: S4 Fig — Manhattan plots show mitotype A. independent and B. mitotype dependent associations, when MIP1 variants were included as covariates. Red lines indicate FDR thresholds at 0.1% for nuclear associations and 5.0% for mitonuclear associations. (TIF) [file pgen.1010401.s019.tif]

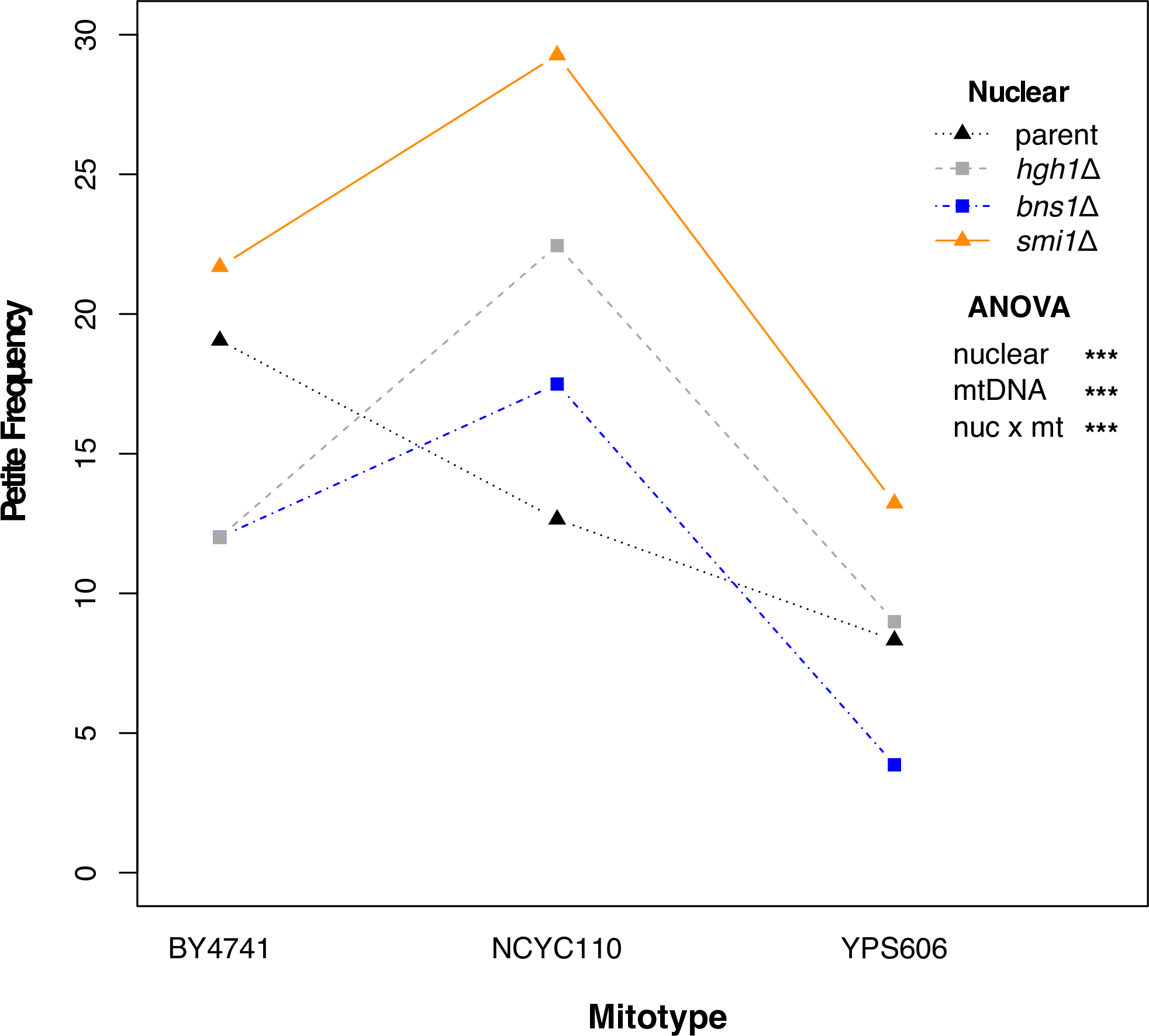

Supplement: S5 Fig — Interaction plot follows petite frequencies for each nuclear genotype paired with different mtDNAs. See S11 Table for ANOVA. Data was collected using the same assay as performed for phenotyping the RCs (with 8–12 replicates) and cannot be combined with the data shown in Fig 6. (TIF) [file pgen.1010401.s020.tif]

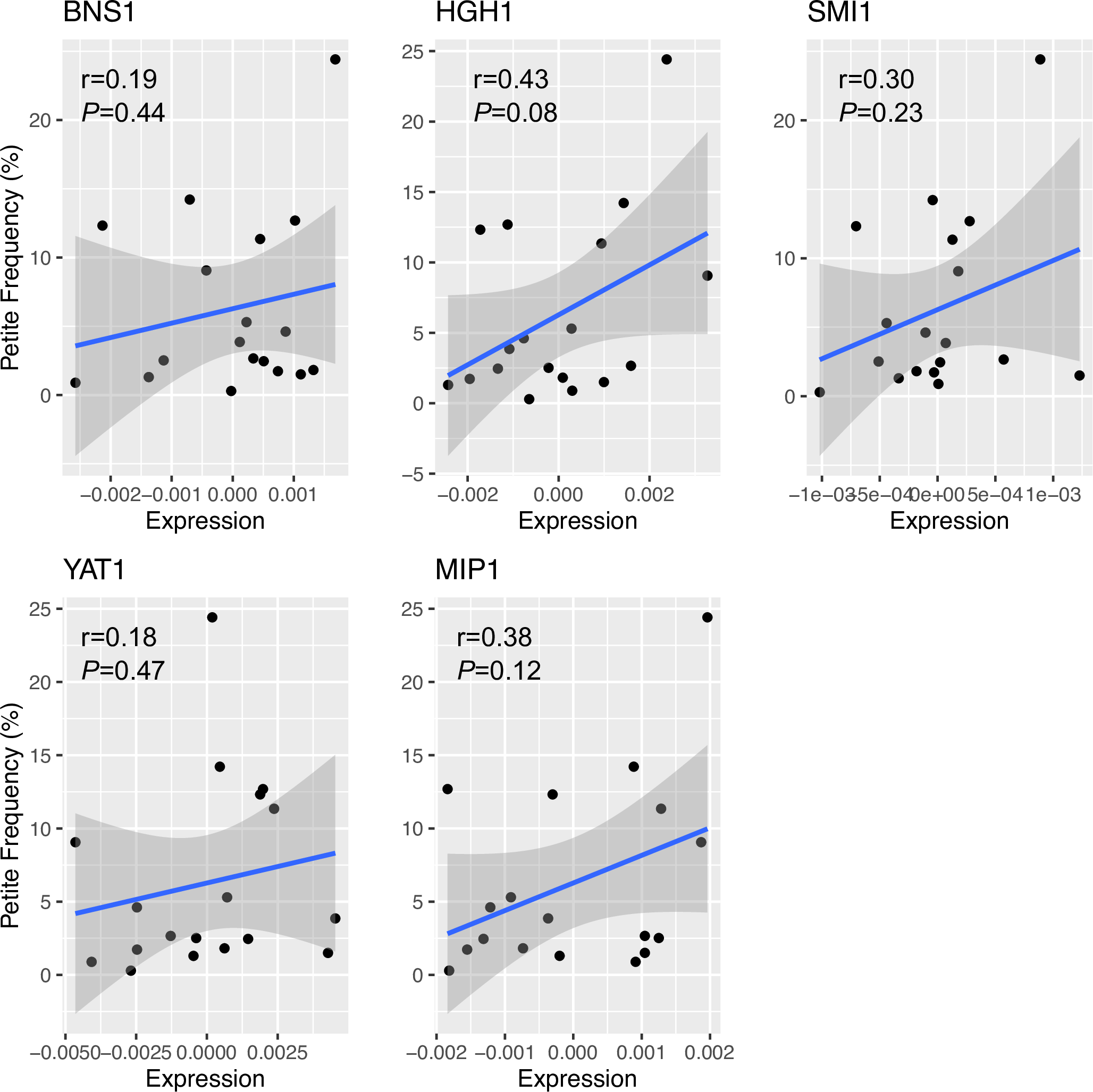

Supplement: S6 Fig — Normalized expression levels (as residuals from regression lines of mRNA levels of each gene compared to a control gene) were plotted against petite frequencies. All genes showed positive correlation with petite frequencies, though no correlation was statistically significant. (TIF) [file pgen.1010401.s021.tif]

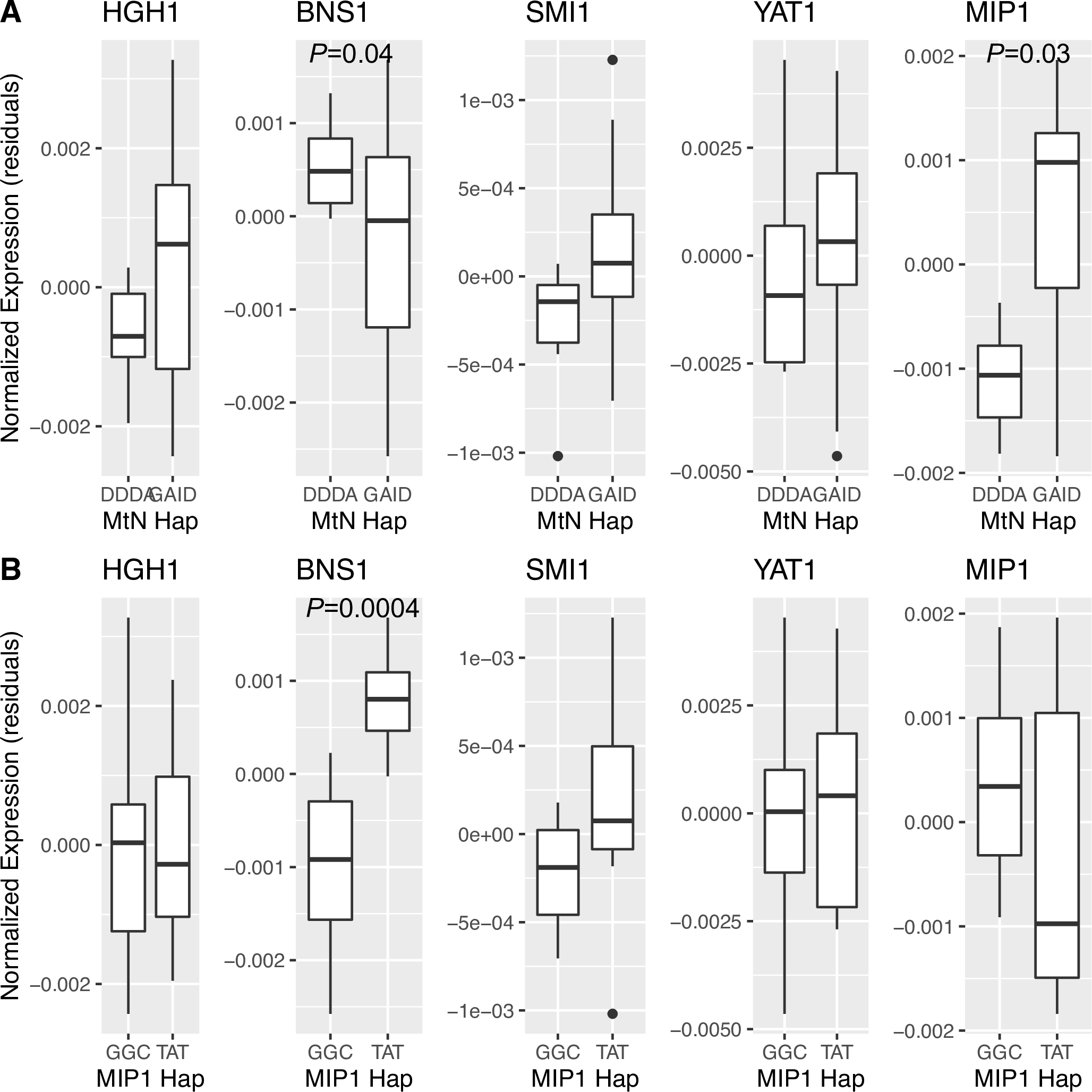

Supplement: S7 Fig — A. Normalized expression levels of each candidate gene separated by haplotypes of A. mitonuclear candidate loci or B. MIP1 loci. The mitonuclear haplotypes represent the SNPs with highest effect sizes for each candidate gene. P values for significant differences are shown. All other comparisons were non-significant. (TIF) [file pgen.1010401.s022.tif]

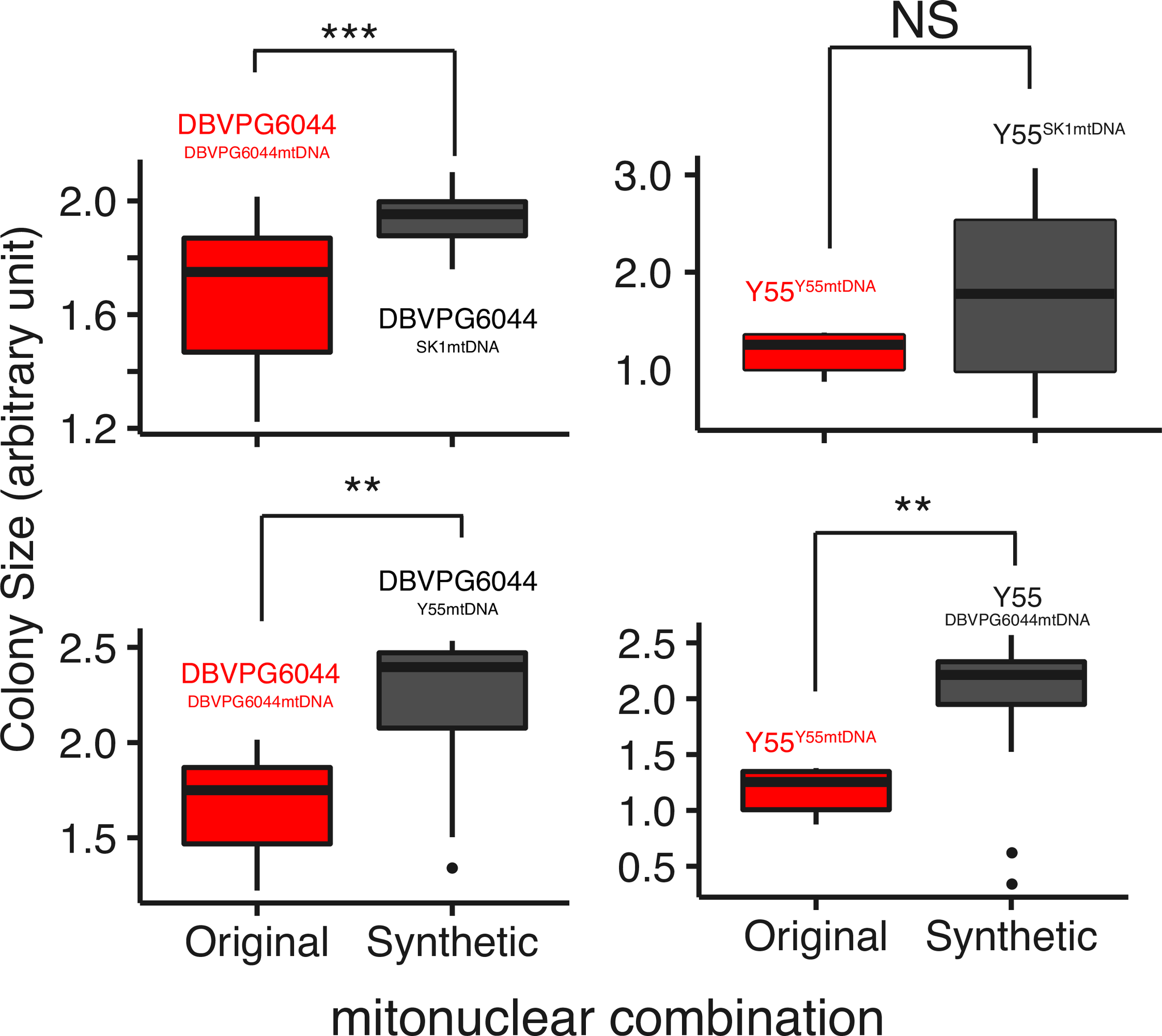

Supplement: S8 Fig — Maximum colony sizes for strains containing original or synthetic mitonuclear genotypes are presented as boxplots. Each synthetic mitonuclear genotype had higher growth, and higher petite frequencies (S1 Fig), than the original mitonuclear genotype. Growth data were from NGUYEN et al. 2020 and collected in the same conditions as the petite assays were performed. * P<0.05, ** P ≤ 0.005, *** P ≤ 0.001. (TIF) [file pgen.1010401.s023.tif]

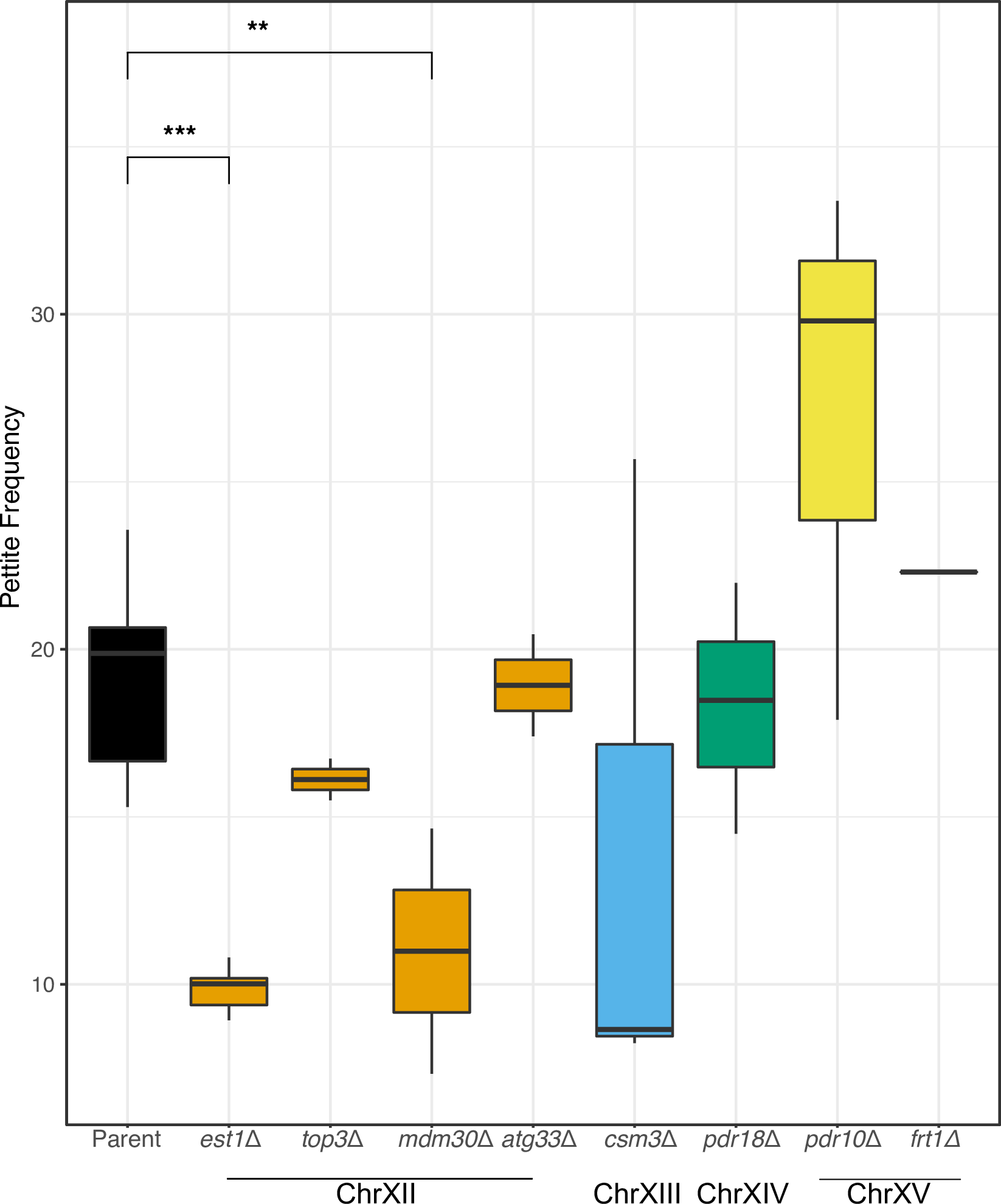

Supplement: S9 Fig — Petite frequencies of strains containing deletions of candidate genes that did not depend on mitotype are shown as boxplots. Significant differences between the petite frequencies of the parental strain and each gene disruption, based on 3 replicates for each strain, are shown. Colors indicate chromosomal location of genes. * P<0.05, ** P ≤ 0.005, *** P ≤ 0.001. (TIF) [file pgen.1010401.s024.tif]
