# Supplementary material for: Mapping mitonuclear epistasis using a novel recombinant yeast population
Source: PLoS Genet. 2023 Mar 29;19(3):e1010401. doi: 10.1371/journal.pgen.1010401 (PMC10085025; doi:10.1371/journal.pgen.1010401)
Supplement: Attachment — Submitted filename: Nguyen_2022.pdf [file pgen.1010401.s025.pdf]

There has been a great deal of interest in mitochondrial genome - nuclear genome (mitonuclear) interactions over the last decade (54 reviews written in the past 10 years). Mitonuclear interactions have been observed in all types of eukaryotes with regard to its effects on evolution, ecology, development, disease, and aging. In a previous manuscript (PMID: 32977769), the authors used a set of 15 isolates and created a series of mtDNA x gDNA combinations. They then examined the growth effects of having the original or novel mitochondrial genomes in each nuclear genome on growth rates under several environmental conditions (media and temperature).

In this manuscript, the authors increase the scale of their previous approach and attempt to define the genes responsible for mitonuclear interactions. They create a set of strains made by crossing 25 wild yeast isolates that had been made mitochondrially homogenous. This process was repeated multiple times to create a population with nuclear genetic diversity but with the same mitochondrial DNA. The mitochondria from the resulting strains were then removed and replaced with two different mitochondrial genomes (using a common yeast genetics trick of mating with karyogamy mutant), resulting in sets of strains to examine how each of the three different mitochondrial genomes interacted with the collection of genetically diverse nuclear genomes. They then measured the frequency of petite formation (loss of mtDNA) from this collection of strains as a quantitative trait.

The main emphasis of the manuscript is a GWAS study that identified alleles which influenced the frequency of petite formation (loss of the mtDNA). Consistent with a potential mitochondrial-nuclear genetic interaction, some of these nuclear alleles were influenced by the mitochondrial genomic context.

The quantitative measurement also led the authors to a couple more general claims: i) a positive correlation between petite frequency and growth rate and ii) a correlation between mtDNA stability and a particular GC-cluster in the mitochondrial genome.

While the manuscript is generally well-written, and the strain collection could be useful to the yeast genetics community, I do not think the level of scientific advancement, as presented, is suitable for PLoS Genetics. Specifically, while the authors identify and try to validate (but see below) genes that are potentially involved in mitonuclear interactions, there is no serious attempt to understand the molecular mechanism involved in these interactions. The nearly two decades of research on this subject has uncovered a wealth of genes that are involved in mitonuclear interactions, and the list proposed by the authors simply adds to those (e.g. PMID:32904421). A real advance would be to demonstrate some mechanistic understanding by which (some of) the previously undocumented genes are involved in this process, beyond what is already known (see below). Consequently I believe this manuscript is better suited for a more specialized journal than PLoS Genetics.

Below are more explicit comments that I hope will help the editor make a decision about the manuscript and allow the authors to improve their study.

1. Critically, the process the authors used to “validate” their GWAS “hits” was not up to the standard in the field (e.g. PMID: 30498022, 19581448, 30504363). To test the genes

potentially involved in mitonuclear interactions, the authors in the present study used deletion alleles from the yeast knock out (YKO) collection, which is based on the lab strain BY4741, with different mitotypes. This has two major issues:

a. It is incorrect to assume that the alleles segregating in wild populations are equivalent to full gene deletion, null alleles (the YKO alleles).

b. The petite frequency of BY4741 (derivative of S288C) is ~50%. This is 5-100X higher than the parental strains used in the screening/analysis (Figures 1 & 5). Consequently the sensitivity of the petite assay for their choice in using this strain to test their candidate “hits” may very likely reduce their ability to see most effects.

Instead, the authors should perform an allele replacement within strains, as is the standard in the field, to test their hypothesis about a gene’s impact on mitonuclear phenotypes (see PMID: 30498022, 19581448, 30504363).

2. Another main claim by the authors is that there is a positive correlation between growth rate and petite frequency. However, in Fig. 7a, they do not include growth rates of each of the strains shown in Fig. 1. Since the correlation is only marginally significant ($P = 0.039$), and this is one of the main claims of the paper, it seems important to increase the power of the correlation by including all of the strains.

3. Similarly, the claim that increased petite frequency is observed when growth rate is increased (by increasing glucose concentration or temperature) is only shown for one of the strains. Since they picked a strain with one of the highest petite frequencies observed in the collection (Y12) to make this point, they should at least establish that this observation holds up for strains with low and intermediate petite formation frequency.

4. The *MIP1* gene, which encodes the mitochondrial DNA polymerase, was the dominant signal in the authors’ GWAS analysis and was also discovered in a similar GWAS mitonuclear interaction screen in yeast published in 2009 (PMID: 19581448). Granted, this earlier GWAS was the first generation of the recombinant mapping methods employed here, it is curious that the authors only cite this paper in passing. Also they do not discuss the fact that this *MIP1* allele has already been observed, nor do they discuss other alleles found in this paper (*MKT1, SAL1*, and *CAT5*). It is worth noting that these alleles have been verified by the community and used to make strains with lower petite frequency (e.g. PMID: 32620756). If they were not discovered in their analysis, it might be worth mentioning (since it suggests a specific interaction with the BY or RM11 genetic backgrounds which have been used in mapping of dozens of studies). Conversely, it may be worth mentioning how these alleles are distributed among the wild isolates they did use, and whether they have any effect on petite frequency in these wild isolates.

5. Related to the previous point, the literature contains a wealth of information about petite-causing alleles (e.g. PMID 32904421 and references therein). Considering that all of the strains used in the submitted manuscript have relatively low petite frequencies

compared to laboratory strains, it would be worth reporting whether any of these alleles were observed in any of the authors' strains. This would provide useful information to the community as to whether these alleles only cause a petite phenotype in the context of the nuclear/mito DNA background of common laboratory strains.

6. Another one of the main claims of the paper is that petite frequency is correlated with the number of M4-type GC clusters (Fig 2B). However, it appears that this correlation is completely determined by the West African strain group, which are outliers relative to the other strains. If this group is removed, is there still a correlation? Again, it seems a direct test of their hypothesis is missing and could be accomplished by moving the GC cluster into a non-GC mitogenome.

7. On line 230, what makes the authors think that the higher number of M4 clusters is a "recent" expansion? There was no apparent phylogenetic analysis to establish a sense of evolutionary time.

8. The authors show, in Fig. 3, Fig. 6a, and Fig. S5, evidence for interactions between the nuclear and mitochondrial genome. As a control, they should perform the same experiment (show the data) for one of the alleles that they claim is *independent* of mitotype (e.g. *MIP1* or *EST1*) and show that the lines are, in fact, parallel (meaning "no interaction" between the genomes).

9. Lines 554 and 555. It seems misleading to claim that "*MIP1* was lower in strains with the low petite frequency alleles and positively correlated with petite frequencies, although not to statistical significance". Especially since the authors also claim (Fig S6) that there is *no* correlation between petite frequency and mRNA expression because the observed positive correlation is not significant. Which is it? This statement should be removed.

10. It is not clear what the rationale was for choosing an FDR < 0.1% with Bonferroni correction for selecting significant "main effects", but an FDR of 5% without Bonferroni correction for selecting mitotype effects. As presented, it appears to be a post-hoc decision to either reduce the number of hits (in the case of 0.1% + Bonferroni), or ensure that at least one hit was found (in the case of 5%). Please state why these cutoff decisions were made.

11. Editorial suggestions:

    a. Use a consistent color scheme between Fig. 5A and Fig. S3

    b. Put the units (mOD/min) on the x axis in Fig. 7. (Also, why not use the more standard OD/hr as the unit of measure?)

    c. L394: "LD" is used without mentioning "linkage disequilibrium"

    d. Parts of the introduction read more like a discussion section (especially paragraph starting on line 77). Consider moving to the discussion or removing.

    e. L176: It would be helpful to state which nuclear background was used.

f. L401: Missing ")" after "(Fig. S7A"

g. Paragraph starting on L438: There appears to be text that is duplicated from earlier in the manuscript (definition of effect size, description of MIP1 allele).
