# Supplementary material for: Mapping mitonuclear epistasis using a novel recombinant yeast population
Source: PLoS Genet. 2023 Mar 29;19(3):e1010401. doi: 10.1371/journal.pgen.1010401 (PMC10085025; doi:10.1371/journal.pgen.1010401)
Supplement: Attachment — Submitted filename: Reviewer Responses.pdf [file pgen.1010401.s026.pdf]

BINGHAMTON UNIVERSITY
STATE UNIVERSITY OF NEW YORK

Harpur College of Arts and Sciences
Department of Biological Sciences
PO Box 6000
Binghamton, New York 13902-6000
607-777-2438, Fax: 607-777-6521

Anita K. Hopper, Academic Editor
Kirsten Bomblies, Section Editor
PLOS Genetics

Jan. 15, 2023

Dear Editors Hopper and Bomblies,

Thank you for the opportunity to submit a revised manuscript titled "Mapping mitonuclear epistasis using a novel recombinant yeast population" to PLOS Genetics for its consideration as a Research Article.

Two independent reviewers of our first submission did not agree regarding whether the work warranted publication in PLOS Genetics. While we were certainly disappointed that one reviewer did not see the novelty or relevance of this work, we absolutely appreciate the time spent by this reviewer and for thoughtful comments from both reviewers and editors. In this resubmission, we better outlined the goals and novelty of our approach, addressed all concerns in this revision and included several additional experiments that help strengthen our findings. As a result, we believe this work makes a significant advance in the field of quantitative genetics by providing new mapping resources and providing insight into pathways influenced by mitonuclear epistasis. I am confident it will be of interest to readers of PLOS Genetics.

Summary of project: In nature, genetic variation will alter the efficiencies of mitonuclear interactions influencing phenotypic differences between individuals and populations, and provide opportunities for natural selection to shape coevolutionary processes. Despite the wide and deep interest in understanding how mitonuclear interactions shape populations, few studies have been successful in mapping mitonuclear loci. In this work, we present a new mapping population of *S. cerevisiae* yeasts created from wild isolates designed explicitly for identifying mitonuclear loci. Our mapping population is unique in that it precisely replaces mtDNA variants (mitotypes) in a multiparent advanced intercrossed recombinant population. We also developed a new association model for mapping loci that specifically incorporates mitonuclear epistasis and does not depend on exact allele frequencies in nuclear genomes between mitotype populations. Our approach is a

significant advance in that it overcomes many of the challenges of mapping epistasis: dominance effects were eliminated, nuclear recombination should reduce LD to provide finer mapping resolution, controlled mtDNA inheritance should reduce unexplained phenotypic variances due to mitochondrial heteroplasmy and (in the case of fungi and some plants) mitochondrial recombination. We validated our yeast mitonuclear recombinant mapping population (MNRC) by mapping alleles contributing to rates of mtDNA loss (*petite* frequencies). In addition to identifying independent effect genes with known involvement in mtDNA stability (eg. *MIP1*), we found that mitonuclear epistasis mapped to genes involved in mitotic growth.

Summary of changes: As suggested by the editors, we revised the text to better frame the goals of the study and address the novelty of our work. We further validated that our statistically mitonuclear associations translated into physiological mitonuclear epistasis by generating additional strains and comparing the effects of knockouts of genes with mitonuclear associations vs. a gene with independent associations (Fig 6B). In the original submission, we showed a positive correlation between growth rates and petite frequencies in a small number of strains. In this revision, we provide growth phenotypes of the entire recombinant collection which resulted in a much more significant correlation between growth and mtDNA stability (Fig 7A). This correlation increased when strong independent associations were taken into account, as would be expected (Fig 7B). We originally showed that increasing growth through media and environmental conditions in one strain correlated with decreased mtDNA stability. Here, we increased this analysis to 6 strains and report a similar trend (Fig 7C). We also highlighted that the high *petite* frequency causing alleles previously identified in the BY background were not identified in our study because this strain was not included as a parent in the recombinant collection (intentionally, as our goal was to look at natural genetic variation), and thus, those alleles were not present. Both reviewers commented that the original manuscript was well-written. We still took advantage of this resubmission to improve and revise the text with small changes for clarity and readability throughout. All substantial changes are shown in the comments to the reviewers and editors, addressed below. Reviewers comments are in **black**; responses in **blue**.

Responses to **Reviewer 2**:

Reviewer #2: Nguyen et al. describe a genetic mapping experiment in yeast to identify mitochondrial, nuclear and mito x nuclear epistatic effects influencing phenotypes and mtDNA genome composition. The authors develop new mapping panels of yeast strains that are particularly focused on mapping mitonuclear interactions, the Mitonuclear Recombinant Collection (MRC). A focal phenotype of the study is the stability of the mitochondrial genome; deletions of mtDNA sequences are common among yeast strains and can be associated with growth phenotypes that are indicative of disease models as well as general yeast fitness.

A screen of natural isolates from around the world revealed significant variation in the incidence of

the petite phenotype (small colonies in a growth assay), which have been, and were shown to be, associated with mtDNA deletions. Subsequent analyses in new stains pairing different mtDNAs with different nuclear backgrounds revealed that nuclear, mtDNA and mito x nuclear interactions explained the variation in growth phenotypes. To map the specific loci involved, the Mitonuclear Recombinant Collection was constructed among 25 nuclear chromosomal isolates engineered to have the same mtDNA, and allowed to recombine for 7 generations. Recombinant haploid descendants of this process were then paired with two alternative mtDNAs generating three different mapping panels harboring extensive nuclear polymorphism on alternative mtDNA backgrounds. Sequencing and phenotyping of these panels allowed gene mapping with GWAS approaches that could detect main and mitonuclear epistatic effects. A number of nuclear loci associated with growth (petite) and mtDNA deletion traits were identified, notably the mitochondrial DNA polymerase gene MIP1. In addition, the molecular aspects of the mtDNA deletions were attributable to GC rich motifs that contribute to deletions. The details of gene and mutation validation were reported.
Overall this panel of strains is a very powerful new mapping tool for mitochondrial genetics, but more generally for considering epistasis mapping questions. The text is clear and the statistical analyses seem fine, as there are strong signals that were followed up with empirical validation. This paper will be of general interest to the PLoS Genetics readership.

Thank you for recognizing the power of this approach and supporting the statistical approaches that we have applied. Most importantly, we appreciate your acknowledgement that this paper should be of general interest to readers of PLoS Genetics.

General Comments:
The different rank orders of QTL effects in the three mapping samples (RC1, 2, 3 - Figure 5) suggest that mtDNA affects the nuclear contribution to the petite frequency, or some kind of mitonuclear interaction. How can one be certain that the allele frequencies at nuclear loci are (precisely) the same in the different RC1, 2, 3 backgrounds? Any difference in these frequencies could alter the power to detect or discover a nuclear variant, which would be interpreted as a mitonuclear interaction when observed across RC1, 2, 3. Perhaps the breeding scheme for the MRC ensures this, but it seems there is plenty of opportunity for sampling drift among alleles in the final (and ongoing culture) of these panels.

We understand the concern about differences in allele frequencies in the three populations. Because we explicitly test for epistasis based on the interaction term from a two-way anova, the model should not yield false positives due to allele frequencies differences. Additionally, we created the mapping populations such that the nuclear genomes are identical across the different mitochondrial haplotypes (to the extent that all strains are present with all mitotypes). The presence or absence of specific mtDNA-nuclear combinations (eg. N1 with M1 and M2 but not M3) will not generate spurious associations. Sometimes epistasis is inferred when statistically significant phenotypic effects are shown with one mitotype but not with another. With that approach, allele frequency differences could allow power differences that produce false evidence for epistasis. Using the interaction term from the two-way anova, as we do, avoids this concern. Yeast strains can be cryogenically preserved, avoiding the concern that new mutations and drift will affect the integrity of the collection over time.

How much of the mtDNA deletion effects were influenced by heteroplasmy level, vs. simple presence absence? This may have been missed in reviewing the manuscript, but was heteroplasmy quantified as relative copy number, percent heteroplasmy, or a +/- trait?

Yeast don’t maintain mtDNA heteroplasmy (for reasons that are not entirely known) and will fix for a single mitotype in just a few mitotic cell divisions. It was previously shown that vast majority of spontaneously occurring non-respiring petite cells are the result of mtDNA deletions. Strains maintaining partial mtDNA deletions (rho-) have a tendence to lose their mtDNAs entirely (rho0) but

phenotypes for rho- and rho0 cells are both respiratory deficient. Thus, we did not quantify heteroplasmy directly and considered *petite* / *grande* as a +/- trait. A description of mtDNA heteroplasmy (and the lack thereof) can found in the Background L136.

Responses to Reviewer 1:

There has been a great deal of interest in mitochondrial genome - nuclear genome (mitonuclear) interactions over the last decade (54 reviews written in the past 10 years). Mitonuclear interactions have been observed in all types of eukaryotes with regard to its effects on evolution, ecology, development, disease, and aging. In a previous manuscript (PMID: 32977769), the authors used a set of 15 isolates and created a series of mtDNA x gDNA combinations. They then examined the growth effects of having the original or novel mitochondrial genomes in each nuclear genome on growth rates under several environmental conditions (media and temperature).

In this manuscript, the authors increase the scale of their previous approach and attempt to define the genes responsible for mitonuclear interactions. They create a set of strains made by crossing 25 wild yeast isolates that had been made mitochondrially homogenous. This process was repeated multiple times to create a population with nuclear genetic diversity but with the same mitochondrial DNA. The mitochondria from the resulting strains were then removed and replaced with two different mitochondrial genomes (using a common yeast genetics trick of mating with karyogamy mutant), resulting in sets of strains to examine how each of the three different mitochondrial genomes interacted with the collection of genetically diverse nuclear genomes. They then measured the frequency of petite formation (loss of mtDNA) from this collection of strains as a quantitative trait.

The main emphasis of the manuscript is a GWAS study that identified alleles which influenced the frequency of petite formation (loss of the mtDNA). Consistent with a potential mitochondrial-nuclear genetic interaction, some of these nuclear alleles were influenced by the mitochondrial genomic context.

The quantitative measurement also led the authors to a couple more general claims: i) a positive correlation between petite frequency and growth rate and ii) a correlation between mtDNA stability and a particular GC-cluster in the mitochondrial genome. While the manuscript is generally well-written, and the strain collection could be useful to the yeast genetics community, I do not think the level of scientific advancement, as presented, is suitable for PLoS Genetics. Specifically, while the authors identify and try to validate (but see below) genes that are potentially involved in mitonuclear interactions, there is no serious attempt to understand the molecular mechanism involved in these interactions. The nearly two decades of research on this subject has uncovered a wealth of genes that are involved in mitonuclear interactions, and the list proposed by the authors simply adds to those (e.g. PMID:32904421). A real advance would be to demonstrate some mechanistic understanding by which (some of) the previously undocumented genes are involved in this process, beyond what is already known (see below). Consequently I believe this manuscript is better suited for a more specialized journal than PLoS Genetics.

We are sorry that the main objective of our work- to provide a novel and powerful mapping resource in yeast that allows for the detection of alleles contributing to mitonuclear epistasis distinct from alleles contributing to phenotypes through direct effects using quantitative genetics - was not more clear. We chose *petite* frequency as a mapping trait to validate our strategy because of the overwhelming amount of research on this trait. This suggested to us that there would be genes with independent effects and others with mitotype-dependent effects. We are embarrassed that our initial submission read as if we did not know about the wealth of data available on *petite* phenotypes. We would like to point out that nuclear mutations leading to the *petite* phenotype do not always occur in ways that are dependent on mitotype; that is they do not demonstrate the mitonuclear epistatic effects that we are after. It is erroneous to think that our results simply add to the long list of genes involved in mitochondrial respiration.

In populations, statistical estimates of epistasis may not translate to physiological epistasis (as defined by Sackton PMID 27419868) when tested in a single genetic background. Mitonuclear

interacting alleles identified through association or other statistical models may not confer phenotypic differences when tested in any particular nuclear background given the large number of quantitative loci that could exist in that chosen background. It is generally believed that effect sizes of epistasis are lower than main effect nuclear loci, and we observed that here in regards to mitonuclear epistasis. This makes it challenging to validate epistatic loci. The excellent suggestions you provided (in particular comment 8, addressed below) helped us to show that the mitonuclear alleles identified through our statistical model do, in fact, impact cellular physiology in ways that are dependent on mitotypes. We think that more detailed mechanistic understandings of how these alleles function is a goal of future studies.

To help readers better understand our goals, we better framed our definition of mitonuclear epistasis and our goals (Background L67-77 and Significance L 654-670). We also rewrote the background section describing the many contributions to understanding the *petite* phenotype in yeast (Lines 135-155). We have also expanded our descriptions of previous studies on mapping of mitonuclear loci (Lines 105-113). We are sorry that this wasn't more clear and hope we have done a better job at placing this work in its proper context.

Below are more explicit comments that I hope will help the editor make a decision about the manuscript and allow the authors to improve their study.

1. Critically, the process the authors used to "validate" their GWAS "hits" was not up to the standard in the field (e.g. PMID: 30498022, 19581448, 30504363). To test the genes potentially involved in mitonuclear interactions, the authors in the present study used deletion alleles from the yeast knock out (YKO) collection, which is based on the lab strain BY4741, with different mitotypes. This has two major issues:

a. It is incorrect to assume that the alleles segregating in wild populations are equivalent to full gene deletion, null alleles (the YKO alleles).

b. The petite frequency of BY4741 (derivative of S288C) is ~50%. This is 5-100X higher than the parental strains used in the screening/analysis (Figures 1 & 5). Consequently the sensitivity of the petite assay for their choice in using this strain to test their candidate "hits" may very likely reduce their ability to see most effects.

Instead, the authors should perform an allele replacement within strains, as is the standard in the field, to test their hypothesis about a gene's impact on mitonuclear phenotypes (see PMID: 30498022, 19581448, 30504363).

8. The authors show, in Fig. 3, Fig. 6a, and Fig. S5, evidence for interactions between the nuclear and mitochondrial genome. As a control, they should perform the same experiment (show the data) for one of the alleles that they claim is independent of mitotype (e.g. MIP1 or EST1) and show that the lines are, in fact, parallel (meaning "no interaction" between the genomes).

Responses to 1 and 8:

It is widely appreciated that statistical models of detecting epistasis do not always translate into predictable phenotypes when looking at individual genotypes. In our association model, we found that nuclear effects (those independent of mitotype) were much stronger than mitonuclear effects. Given that the BY nuclear background for the knockout collection carries a number of alleles that greatly increase influence petite frequencies (in *MIP1*, *CAT5, SAL1*), it was possible that mitonuclear interactions would be swamped by the nuclear effects in the knockout collection. We were, however, able to detect the mitonuclear interactions in the knockout collection and validate the effects of the specific nuclear genes we found from the GWAS. This demonstrates the success of our model and our mapping population (and addresses concern 1b). We agree with the editors that allele replacements are not likely to change our findings, and it would not be clear which strains should be chosen for this type of analysis. That said, directed allelic replacement may prove useful in future studies aimed at understanding the molecular mechanism of observed interactions.

We loved the idea of showing what happens in deletions of genes with mitotype-independent associations. We introduced 2 new mitotypes into the *est1*Δ knockout strain and estimated petite

frequencies (we could not do this with *mip1*Δ, as a knockout is ρ^0, and thus *petite* frequencies cannot be determined). We found that the *petite* frequencies of an *est1*Δ roughly parallel those of the parental background, as would be expected for genes with main nuclear effects, while the deletions of mitonuclear-associated loci *hgh1*Δ and *yat1*Δ show different effects. We think this nicely validates that our model successfully detected nuclear vs. mitonuclear loci. This data can be seen in the updated Fig 6B and in the following text:

Results L379-388:
"The mitonuclear interactions detected in our GWAS model may be dependent on particular nuclear backgrounds. Still, mitonuclear interactions in the reference strain background (BY4741) were readily observed; the GC-rich mitotype led to higher *petite* frequencies in the *hgh1*Δ and *yat1*Δ deletion strains, whereas the same mitotype led to a lower *petite* frequency in the parental background (**Fig 6B**). This is in contrast with the mitotype independent effect observed in *est1*Δ strains, whose *petite* frequencies roughly paralleled the parental background. Two-way ANOVAs showed highly significant mitonuclear interactions when comparing the parental strain to *hgh1*Δ and *yat1*Δ deletion strains with each mtDNA comparison (P<0.001, **Table S10**)."

and

Results L494-502:
"In the parental background of the deletion collection, *petite* frequencies of an *est1*Δ were similarly reduced when different mitotypes were introduced (**Fig 6B**), as would be expected for mitotype-independent associations. Two-way ANOVAs comparing *est1*Δ and parental strains with different mtDNAs showed very strong nuclear and mtDNA effects ($P<2.2 \times 10^{-16}$) but weak mitonuclear effects (P<0.05, not significant after Bonferonni correction **Table S10**). These weak mitonuclear effects are likely driven by low *petite* frequencies in the non-parental mitotypes (especially the YPS606 mitotype). As petite frequencies approach zero, differences between nuclear genotypes will appear non-additive."

2. Another main claim by the authors is that there is a positive correlation between growth rate and petite frequency. However, in Fig. 7a, they do not include growth rates of each of the strains shown in Fig. 1. Since the correlation is only marginally significant (P = 0.039), and this is one of the main claims of the paper, it seems important to increase the power of the correlation by including all of the strains.

This is a valid concern. We determined growth rates in liquid cultures for the parental strains shown in Fig 1, but found that many of them flocculated. Therefore, we phenotyped the entire RC1 and RC2 collections for growth differences using colony arrays. Across RC1 and 2, we found that growth (differences in colony sizes) weakly correlated with *petite* frequencies but with much greater significance that what was observed in the original analysis of 10 strains. To test if nuclear effects masked the growth phenotypes potentially conferred by mitonuclear epistasis, we partitioned the strains into those with high and low *petite* frequency *MIP1* alleles with the highest effect size and found that the correlations between growth and *petite* frequencies increased in strains with the low *petite* frequency *MIP1* allele. We think these new data provide better support that *petite* frequencies correlate with mitotic growth phenotypes.

We removed the original Fig 7A and replaced with new figures showing the positive correlations between growth differences of the RC1 and RC2 (Fig 7A) and RC2 with the MIP1-C allele (Fig 7B). We removed the text describing growth rates of the 10 parental strains and replaced as follows:

Results L431-437
"We observed a significant positive correlation between the growth (measured as colony sizes on solid media) of RC1 and RC2 strains with their petite frequencies (r=0.16, P<0.002, **Fig 7A**).

Removal of the one outlier did not influence this correlation. This effect is strongly influenced by strains with the low petite frequency allele of *MIP1* at position 943237 (*MIP1*-C) paired with the RC2 mitotype (r=0.37, P<0.001, **Fig 7B**). Petite frequencies of strains with the high petite frequency allele (*MIP1*-T) did not show a significant correlation with growth (P>0.05)."

Methods L703-707
"Growth differences of RC strains were determined by spotting cells in high densities arrays onto solid CSM media using a BM3-BC colony processing robot (S&P Robotics) following the methodology described in [15] with 5-12 replicates per strain. The difference between maximum and minimum colony size formation at 30C was used as a proxy for growth."

3. Similarly, the claim that increased petite frequency is observed when growth rate is increased (by increasing glucose concentration or temperature) is only shown for one of the strains. Since they picked a strain with one of the highest petite frequencies observed in the collection (Y12) to make this point, they should at least establish that this observation holds up for strains with low and intermediate petite formation frequency.

We increased the number of strains in these analyses from one to six, including strains with low and intermediate petite frequencies from different ancestral populations. We found that growth rates (estimated in liquid cultures) increased from low to high sugar at 30C, and from high sugar at 30C to 37C. Similarly, we noted an increase in petite frequencies following this trend for strains from Sake and Malaysian populations and in 1 of the 2 North American isolates tested. The North American with the lowest petite frequency, and both Wine/European strains showed a slight increase in petite formation frequencies at high temperature, but overall maintained close-to-zero petite freq. averages. This suggests that there are additional genetic factors that can stabilize mtDNAs.

We show these results in an updated Fig 7C, and in the following text:
Results L438-451:
"To investigate a potential relationship between mitotic growth and mtDNA stability, we compared the growth rates of six wild yeast isolates in different environmental conditions and their corresponding *petite* frequencies (**Fig 7C**). Growth rates of each strain increased between growth in media containing low (0.2%) and high (2.0%) glucose concentrations and high temperature (**Fig 7C**, top panel). The increases in Vmax were greatest between 30C and 37C. *Petite* frequencies (bottom panel) also showed increases between 30C and 37C. Overall, conditions that increased growth rates increased petite frequency, although the petite frequency increases were not necessarily proportional to Vmax increases. The two strains with Wine/European ancestries maintained low *petite* frequencies even at high temperatures. It is likely that ancestral polymorphisms in these strains have strong nuclear effects that stabilize mtDNAs. Still, these data suggest that increasing mitotic growth rates can lead to a decrease in mtDNA stability, possibly exposing a fitness tradeoff between rapid cell growth and deletions in mtDNA."

4. The MIP1 gene, which encodes the mitochondrial DNA polymerase, was the dominant signal in the authors' GWAS analysis and was also discovered in a similar GWAS mitonuclear interaction screen in yeast published in 2009 (PMID: 19581448). Granted, this earlier GWAS was the first generation of the recombinant mapping methods employed here, it is curious that the authors only cite this paper in passing. Also they do not discuss the fact that this MIP1 allele has already been observed, nor do they discuss other alleles found in this paper (MKT1, SAL1, and CAT5). It is worth noting that these alleles have been verified by the community and used to make strains with lower petite frequency (e.g. PMID: 32620756). If they were not discovered in their analysis, it might be worth mentioning (since it suggests a specific interaction with the BY or RM11 genetic backgrounds which have been used in mapping of dozens of studies). Conversely, it may be worth mentioning

how these alleles are distributed among the wild isolates they did use, and whether they have any effect on petite frequency in these wild isolates.

The BY or S288C reference strains contain alleles in *MIP1*, *SAL1* and *CAT5* that increase *petite* frequencies (and a variant of *MKT1* that is thought to stabilize mtDNA). These reference strains were *not* part of our parental panel for the MNRC, and therefore their unique genetic variants should not exist in the mapping population. A derivative of W303 (which is ~85% related to S288C and BY) was included as one of the mat alpha haploids in the parental strains, however we noted that the auxotrophic markers in this strain were completely absent in the recombinant progeny after just 1 round of meiosis, suggesting that this genotype was lost during strain construction. We inspected the SNP tables and confirmed the absence of the BY alleles. We did not identify them during our mapping because they were not present. We did identify 3 (and to our knowledge, previously undescribed) *MIP1* variants (predicting G50D, T540M and H541N changes). Given that *MIP1* is well established with its involvement in mtDNA replication and that the goal of the paper was to identify mitonuclear epistatic alleles, we did not further explore these mutations.

We updated the text to point out that the reference strain, and it's unique alleles, are not included in our study. We apologize for not recognizing that we should have drawn attention to this earlier and hope our new text clears up any confusion.

Results L479-483
"In addition to the *MIP1* G50D missense mutation in the mitochondrial DNA polymerase, MIP1, two additional *MIP1* missense variants (T540M and H541N) were also significant. These alleles are different from the previously characterized *MIP1-661A* allele that leads to high petite frequency in the reference strain [62]."

Discussion L604-617
"Loci with main effects for mtDNA stability detected by our model interact with mtDNA through non-specific binding. In the MNRC, missense alleles of the mtDNA polymerase, *MIP1*, predicting G50D, T540M and H541N amino acid changes, had the strongest effects on *petite* frequencies. It is important to note that only one relative of the yeast reference strain was included in the parental collection of the MNRC, and that selection against some of the auxotrophic markers in this genotype led to its loss during construction of the recombinant collection. Given that, and that association testing is greatly influenced by allele frequencies, the high-petite frequency *MIP1*-661A allele and other petite-influencing alleles of *CAT*5, *SAL*1 and *MKT*1 found in many S288c-derived strains [55] were not detected in our analyses. We did not explore the mechanisms of these novel *MIP1* alleles, though other alleles in *MIP1* are known to influence mtDNA stability [55,92,93]. Our findings suggests that additional naturally occurring *MIP1* variants contribute to basal differences in mtDNA stability in populations, though the frequencies of these alleles in yeast isolates has not yet been determined."

Results L270-274
"Only one laboratory strain (W303, derived from the reference strain) was included as a parent strain. We noted that very few progeny after one round of meiosis contained the selectable markers found in this strain, suggesting that this genotype was quickly lost from the collection."

5. Related to the previous point, the literature contains a wealth of information about petite-causing alleles (e.g. PMID 32904421 and references therein). Considering that all of the strains used in the submitted manuscript have relatively low petite frequencies compared to laboratory strains, it would be worth reporting whether any of these alleles were observed in any of the authors' strains. This would provide useful information to the community as to whether these alleles only cause a petite phenotype in the context of the nuclear/mito DNA background of common laboratory strains.

As explained about, we updated the Background (Lines 135-153) to better draw attention to the large body of work investigating *petite* phenotypes in the reference strains. While it would be a worthwhile endeavor to examine the wild isolates for every *petite*-causing variant, it does not fit within the scope of this project.

6. Another one of the main claims of the paper is that petite frequency is correlated with the number of M4-type GC clusters (Fig 2B). However, it appears that this correlation is completely determined by the West African strain group, which are outliers relative to the other strains. If this group is removed, is there still a correlation? Again, it seems a direct test of their hypothesis is missing and could be accomplished by moving the GC cluster into a non-GC mitogenome.
Yes, the M4-type GC cluster correlation is determined solely by the West African strain group. We updated the text as follows and put a caveat that other features may be influencing mtDNA instability:

Results L210-213
"This particular cluster appears to be expanding in the mtDNAs of strains with West African lineages [76] and **these strains** explain the observed correlation. **It is also possible that an unidentified feature in the West African mtDNAs contributes to their instability**."

Our correlative findings of the main effects of mitotypes generate a new hypothesis that M4-clusters (or the placement of those clusters) leads to mtDNA stability that can be tested in future studies. The suggestion to test this hypothesis by introducing M4 clusters into a novel mitotype is intriguing. One would need to introduce one or more M4 clusters into precise positions within a mitochondrial genome lacking these clusters and test for their effects on mtDNA stability. While directed mitochondrial mutations are sometimes possible, they are not routine and require biolistic transformations, homologous recombination and selection methods to ensure that the new mitochondrial variant has been introduced. Personal experience suggests that repeated sequences (such as GC clusters) in mtDNA frequently lead to unstable mtDNA insertions as they would be particularly prone to recombination errors and suggest that this approach will likely fail. Other approaches could include the introduction of synthetically created mtDNAs into a rho0 strain, or using a mtDNA-directed CRISPR, though neither approach has been successful or adopted.

7. On line 230, what makes the authors think that the higher number of M4 clusters is a "recent" expansion? There was no apparent phylogenetic analysis to establish a sense of evolutionary time.

We have not performed a phylogenetic analysis to establish an evolutionary time. Therefore the word "recent" was replaced with "population-specific".

Results L 246
"Given the **population-specific** expansion of the destabilizing M4 clusters within the West African strains, this observation suggests that selection for mitonuclear interactions that stabilize mtDNAs has occurred quickly in ways that are strain-specific."

9. Lines 554 and 555. It seems misleading to claim that "MIP1 was lower in strains with the low petite frequency alleles and positively correlated with petite frequencies, although not to statistical significance". Especially since the authors also claim (Fig S6) that there is no correlation between petite frequency and mRNA expression because the observed positive correlation is not significant. Which is it? This statement should be removed.

We agree that this was an improper statement. This claim as been removed.

10. It is not clear what the rationale was for choosing an FDR < 0.1% with Bonferroni correction for selecting significant "main effects", but an FDR of 5% without Bonferroni correction for selecting

mitotype effects. As presented, it appears to be a post-hoc decision to either reduce the number of hits (in the case of 0.1% + Bonferroni), or ensure that at least one hit was found (in the case of 5%). Please state why these cutoff decisions were made.

It was expected that the main effects would be greater than epistatic effects. The Manhattan plot of the mitonuclear associations shows a distinct structure that looks very different from "noise", even though the effect sizes are much lower than those observed in the main effect associations and two-way interactions are more difficult to detect than main effects as *P*-values are expected to be less significant due to differences in degrees of freedom in the statistical models. Thresholds defining statistical significance for GWAS studies can be somewhat arbitrary and the goal is to identify as many real associations as possible while limiting the potential for false positives. We chose different thresholds for both components of the association model based on the quality of the Manhattan plot structures, allowing us to focus on "hits" originating from peaks, rather than baseline noise. Importantly, we were able to verify that the associated loci conferred phenotypes consistent with both nuclear effects and mitonuclear epistasis. To this end, it is not critical to stick to a single significance threshold.

11. Editorial suggestions:
We thank the reviewer for pointing out these inconsistencies and errors.

a. Use a consistent color scheme between Fig. 5A and Fig. S3
Fig S3 was updated for consistent coloring.

b. Put the units (mOD/min) on the x axis in Fig. 7. (Also, why not use the more standard OD/hr as the unit of measure?)
This figure was updated to include mOD/min on the plot directly. Our plate reader provides an analysis of Vmax from growth curves reported as mOD/min. Changing the units would have no effect on our conclusions and so we have kept those units here.

b. L394: "LD" is used without mentioning "linkage disequilibrium"
We defined linkage disequilibrium at this position (now L417).

d. Parts of the introduction read more like a discussion section (especially paragraph starting on line 77). Consider moving to the discussion or removing.
The introduction has been revised as described above.

e. L176: It would be helpful to state which nuclear background was used.
This genetic background was included in the legend for Fig 2C, and is now included in the main text (L190 "..into a common nuclear background (Y55)..")

f. L401: Missing ")" after "(Fig. S7A"
fixed (now L424)

g. Paragraph starting on L438: There appears to be text that is duplicated from earlier in the manuscript (definition of effect size, description of MIP1 allele).
Thank you for catching this. We revised the text of this section to avoid duplication. The relevant text (now beginning on L478) now reads "Effect sizes (table S9) were used to help identify causative loci."

I declare that this manuscript is original, has not been published before and is not currently being considered for publication elsewhere. All authors have approved the contents of this paper and have agreed to the PLOS Genetics submission policies. Thank you for your consideration.

On behalf of all co-authors,

Heather L. Fiumera, Corresponding Author
Associate Professor
Binghamton University, 4400 Vestal Pkwy E, Binghamton, NY 13902
hfiumera@binghamton,edu
cell: 1-607-321-3711
