# Supplementary material for: Mapping mitonuclear epistasis using a novel recombinant yeast population
Source: PLoS Genet. 2023 Mar 29;19(3):e1010401. doi: 10.1371/journal.pgen.1010401 (PMC10085025; doi:10.1371/journal.pgen.1010401)
Supplement: Attachment — Submitted filename: Response to Reviewer.pdf [file pgen.1010401.s027.pdf]

BINGHAMTON UNIVERSITY
STATE UNIVERSITY OF NEW YORK

Harpur College of Arts and Sciences
Department of Biological Sciences
PO Box 6000
Binghamton, New York 13902-6000
607-777-2438, Fax: 607-777-6521

Anita K. Hopper, Academic Editor
Kirsten Bomblies, Section Editor
PLOS Genetics

Mar. 3, 2023

Dear Editors Hopper and Bomblies,

Thank you for the opportunity to submit a revised manuscript titled "Mapping mitonuclear epistasis using a novel recombinant yeast population" to PLOS Genetics for its consideration as a Research Article.
We are glad that our work and recombinant mapping population is viewed as valuable and could lead to new genetic mechanisms of mitonuclear interactions. We are happy that the extensive concerns of Reviewer 1 have been addressed to satisfaction with our 2nd resubmission. We misunderstood the concern of Reviewer 2 (David Rand) regarding differences in nuclear genotypes, and hopefully have addressed that now.
I remain confident that this study will be of interest to readers of PLOS Genetics.

Summary of changes:. Reviewer comments are in **black**; responses in **blue**.
Responses to Reviewer 2:
Reviewer #2: The comments of reviewer 2 focused on potential spurious effects of allele frequencies differences between the recombinant populations that might leave a signal of QTLs for mitonuclear interactions. The statement that the collection of recombinant nuclear haplotypes is 'identical' across mtDNA backgrounds is taken on faith that the yeast genetic cytoplasmic replacement are clean. It would still help to address this by testing whether the evidence for nuclear x nuclear epistatic effects do not differ across the different mtDNA backgrounds. Any such variation would/could be statistically identified as a mitonuclear effect based on the 2-way anova approach. There certainly must be strong nuclear-nuclear epistatic effects on the phenotype measured (does the effect of nuclear SNP x depend on the state of nuclear SNP y elsewhere in the genome; this could be modeled with jackknife samples of the RC2 and RC3). If there are big differences between the population/samples in these effects that would requires some additional explanation in relation to the mtDNA epistases.

We misunderstood the concern about allele frequency difference (and thought it was a confusion on how the recombinant collection was created). My apologies! You are absolutely correct that differences in allele frequencies between the 3 mitotype populations could falsely identify a nuclear x nuclear epistatic effect as a mitonuclear effect and that is not something that we had considered. We did validate that the mitonuclear associations identified through our GWAS study had physiological mitonuclear effects on mtDNA stability in knockout strains, so we are confident that these are not false associations. Still, allele frequency because of novel mutations in any one strain, or more likely, because of differences in the numbers of isonuclear strains between each mitotype population could be problematic. We calculated the allele frequencies for each of SNP in each of the mitotype population (included as Table S15). For the mitonuclear-significant associated SNPs for petite frequencies, allele frequency differences between each population ranged from 0.1 to 12.4%. To make sure that nuclear x nuclear epistasis did not contribute to these associations, we performed additional association analyses in RC1 using the model *petite frequency ~ nuclear* SNP_i * *mitonuclear-associated* SNP_j and found that the lowest FDR value was 0.08. We think this shows that nuclear x nuclear interactions did not sway the findings we report here.

We liked the idea of resampling approaches to test for false identification of mitonuclear SNPs due to allele frequency differences. Any differences in associations when resampling from RC2 and RC3 through the suggested jackknife approach could be indicative of higher order nuclear x nuclear x mtDNA interactions (effectively a mitonuclear interaction). Instead, we took a different resampling approach: We randomly sampled RC1 strains (and phenotypes and covariates) to match sample sizes in RC2 and RC3, and then performed a GWAS on the RC1 strains and the subsets (mock RC2 and RC3 populations) looking for “mitonuclear” interactions. This was repeated 100 times. Any significant “mitonuclear” associations would be due to nuclear x nuclear interactions as only 1 mitotype was present in these permutations. Across the ~2.5×10^7 tests, we found 30 (~0.001%) with an FDR of <0.05, demonstrating that reviewer 2 was correct in that allele frequency difference could lead to false mitonuclear associations. In these simulations, however, the proportion of the true null hypotheses (pi0) for the distribution of test results was estimated (via qvalue) to be 1; ie- 100% of null hypotheses are estimated to be true (and none of the “significant” associations in these resamplings are estimated to be true). In contrast, the pi0 value for the GWAS presented in this manuscript was 0.6; ie- some of the significant associations are likely to be true, as we confirmed by replacing mitotypes in knockout strains.

We are grateful that you drew attention to this as a potential problem. For future uses of this mapping tool, we will expand the mitotype populations so that allele frequencies are not different due to strain numbers (and are in the process of doing so). There is always a potential for novel

mutations in one or more strain. We think these would most likely have minor effects on allele frequencies and would require large effect sizes in order to alter QTL peaks. We do have biological replicates of most of the recombinant strains in RC2 and RC3 (ie we isolated multiple independent mtDNA recipients during karyogamy deficient matings). These can be included in future analyses while factoring in duplication of genotypes (that could falsely increase associations). Alternatively, all significant mitonuclear associations should be followed up by looking for nuclear x mitonuclear-associated SNP interactions in each mitotype population.

To draw attention to this potential issue, we added the following text to the discussion in the section describing the MNRC.
Lines 543-554:

"We did not test for nuclear-nuclear epistasis across the recombinant collections. Allele frequency differences due to the different numbers of strains in each mitotype population, or novel mutations during strain construction, could theoretically falsely identify nuclear-nuclear interactions as a mitonuclear interactions. Of the three SNPs with the most significant mitonuclear associations (Chr7 positions 949775, 951886 and 871605), the largest allele frequency difference between mitotype populations was 12.4% (Table S15). We looked for evidence of nuclear x nuclear epistasis at these SNPs by performing additional association analyses using RC1 (*petite frequency ~ nuclear* SNP_i * *mitonuclear-associated* SNP_j). We found no evidence of nuclear x nuclear epistasis at these loci (with the lowest FDR = 0.08). Balancing the strain numbers in each mitotype population and testing for nuclear x nuclear interactions for candidate SNPs will reduce the likelihood of misidentifying mitonuclear interactions in future studies."

I declare that this manuscript is original, has not been published before and is not currently being considered for publication elsewhere. All authors have approved the contents of this paper and have agreed to the PLOS Genetics submission policies. Thank you for your consideration.

On behalf of all co-authors,

Heather L Fiumera

Heather L. Fiumera, Corresponding Author
Associate Professor
Binghamton University, 4400 Vestal Pkwy E, Binghamton, NY 13902
hfiumera@binghamton,edu
cell: 1-607-321-3711
